# *Costus afer* Protects Cardio-, Hepato-, and Reno-Antioxidant Status in Streptozotocin-Intoxicated Wistar Rats

**DOI:** 10.1155/2018/4907648

**Published:** 2018-11-25

**Authors:** Armelle D. Tchamgoue, Lauve R. Y. Tchokouaha, Nole Tsabang, Protus A. Tarkang, Jules-Roger Kuiate, Gabriel A. Agbor

**Affiliations:** ^1^Centre for Research on Medicinal Plants and Traditional Medicine, Institute of Medical Research and Medicinal Plants Studies, P.O. Box 13033, Yaoundé, Cameroon; ^2^Department of Biochemistry, University of Dschang, P.O. Box 67, Dschang, Cameroon

## Abstract

Medicinal plants are efficient modulators of oxidative stress associated with diabetes mellitus. This study evaluated the cardio-, reno-, and hepato-antioxidant status of hydroethanolic extract of* Costus afer* on streptozotocin-intoxicated diabetic rats. Experimental animals were daily administered with hydroethanolic extract of* C. afer* by oral intubation for eight weeks (60 days), after which the levels of catalase (CAT), superoxide dismutase (SOD), glutathione (GSH), and lipid peroxidation marker (MDA) were evaluated in the heart, liver, and kidney homogenates. Plasma biochemical parameters such as aspartate aminotransferase (AST), alanine aminotransferase (ALT), alkaline phosphatase (ALP), lactate dehydrogenase (LDH), total protein, creatinine, and urea were determined. Meanwhile, parts of the heart, kidneys, and liver were histopathologically examined. Streptozotocin administration induced toxicity in the cardiac, hepatic, and renal tissues by stimulating significant increases (p<0.05) in the levels of CAT and SOD, GSH, and MDA. Similarly, significant increases (P<0.05) in the levels of ALT, AST, urea, and total protein were observed in streptozotocin treated rats, whereas decreases were observed in the levels of ALP, LDH, and creatinine. Following the treatments with* C. afer* hydroethanolic extract prevented the effect of streptozotocin by maintaining the tissue antioxidant status (CAT, SOD, GSH, and MDA) and the plasma biochemical parameters (AST, ALT, ALP, LDH, creatinine, and urea) towards the normal ranges. The histopathological examination revealed hepatovascular congestion and leucocyte infiltration as well as renovascular congestion, glomerulosclerosis, and tubular clarification in the untreated diabetic control and their absence in the group of animals treated with a high dose of* C. afer* extract. The findings of the present investigation suggest that* C. afer* possesses antioxidant activities capable of regulating drug induced tissue damage.

## 1. Introduction

Reactive oxygen species (ROS) are implicated in the development of a number of diseases such as atherosclerosis, diabetes, cancer, AIDS, hepatitis, and other degenerative diseases [1, 2]. In the diabetic state there is an impairment of pancreatic islets function resulting in the accumulation of circulating glucose concentration associated with an increase in reactive oxygen species [3]. The increase in reactive oxygen species over power endogenous antioxidant defense network resulting in oxidative stress may lead to defective insulin gene expression and insulin secretion as well as increased apoptosis [3, 4]. The increase in oxidative stress in diabetic condition may also lead to other complications such as retinopathy and nephropathy [5]; that is the reason why vitamins E and C (antioxidant) have been considered to be associated with diabetic treatment [6, 7].

One of the main chemical agents in use today for the induction of experimental diabetes in animals is streptozotocin (STZ) [8]. STZ alters carbohydrate and lipid metabolism, hepatic and renal thiobarbituric acid reactive substances (TBARS) levels, hepatic reduced glutathione (GSH) concentration, superoxide dismutase (SOD), and catalase (CAT) activities [9] in experimental rats through free radical mechanism.

Plants often contain bioactive compounds which possess antioxidant properties such as tocopherols (vitamin E), carotenoids, ascorbic acid, flavonoids, and tannins [10, 11]. Considering that oxidative stress may be a causative factor or consequence of diabetes and its complications, we suggest that antioxidant action may be an important property of plant medicines for the management of diabetes. Hence, plants may just serve as the exogenous antioxidants source needed to maintain endogenous antioxidant status threatened by oxidative stress.* C. afer* may provide the much needed antioxidant substances since it earlier demonstrated an in vitro antioxidant capacity and inhibited carbohydrate metabolizing enzymes activity [12]. Meanwhile, extracts of leaves, stem, and rhizome of* C. afer* showed good activities on carbohydrates tolerance tests and modulated glucose uptake by yeasts cells [13].

In the present study, the effects of the hydroethanolic extract of* C. afer* were evaluated for their potential in modulating cardio-hepato-reno toxicity induced by streptozotocin intoxication in Wistar albino rats.

## 2. Material and Methods

### 2.1. Preparation of Plant Material

Leaf sample of* Costus afer* (A. Rich.) was collected fresh from its natural habitat in Yaoundé, Cameroon, with the assistance of an Ethnobotanist, Dr. Tsabang Nole. The leaves were rinsed with tap water, chopped into small pieces, air-dried at room temperature, and then crushed to fine powder using a blender. The air-dried and powdered sample was extracted by maceration twice with a mixture of hydroethanolic solvent (80:20). Hydroethanolic extract was concentrated to reduce volume using a rotary evaporator and finally powdered by evaporating the remaining solvents in a hot air oven at 40°C. The extract was stored at -20°C until further use.

### 2.2. Animals

Male Wistar rats produced by the Institute of Medical Research and Medicinal Plants Studies (IMPM) weighing 160-220g were acclimatized at the temperature of 23 ± 2°C with controlled humidity conditions (50 - 55%) at 12 h light and dark cycle. The rats were kept in polypropylene cages and were fed with standard feed and water was provided* ad libitum* to all experimental animals.

### 2.3. Induction of Experimental Diabetes

Rats were fasted for 16 h before the induction of diabetes with STZ (Sigma Chemical co., St Louis, MO, USA). Animals were injected intraperitoneally with a freshly prepared solution of STZ (dose of 55 mg/kg of body weight in cold saline water). The rats were allowed to drink 5% glucose solution overnight to overcome hypoglycemia. Diabetic state was confirmed on the seventh day and rats with serum glucose levels > 250 mg/dl were considered to be diabetic. The treatments with plant extract and standard drug (metformin 250 mg/kg) were then started after diabetic conditions established and lasted 60 days (eight weeks). In order to detect any changes in body weight, animals were weighed every week during the treatment.

The rats were divided into six groups comprising six animals each.

Group 1: Normal control, received distilled water;

Group 2: Diabetic control, received distilled water;

Group 3: Diabetic rats treated with 250 mg/kg of hydroethanolic extract of* C. afer*;

Group 4: Diabetic rats treated with 500 mg/kg of hydroethanolic extract of* C. afer*;

Group 5: Diabetic rats treated with 1000 mg/kg of hydroethanolic extract of* C. afer*;

Group 6: Diabetic rats treated with metformin (250 mg/kg).

#### 2.3.1. Collection of Blood and Tissues Samples

After 60 days of treatment, rats were sacrificed under anesthesia of diethyl ether. Blood samples were collected from the carotids veins and centrifuged at 3000 rpm for 15 min. The plasma was separated and used for the evaluation of biochemical parameters. The liver, kidneys, and heart were harvested, cleaned with ice cold saline, and weighed. One part of these organs was preserved in 10% formalin for histological studies. The other part served for oxidative stress markers analysis.

#### 2.3.2. Preparation of Tissue Homogenate

Organs (heart, liver, and kidneys) were excised, freed of surrounding tissues, blotted with clean tissue paper, weighed, and homogenized in ice phosphate buffer (0.1 M, pH 7.4), to obtain 10% homogenate (w/v). The homogenates were centrifuged at 3000 rpm for 10 minutes to obtain the supernatants kept frozen overnight at – 20°C before being used in the assays.

### 2.4. Biochemical Parameters

The plasma transaminases (ALT, AST), alkaline phosphatase (ALP), lactate dehydrogenase (LDH), urea, and creatinine levels were assayed using the corresponding commercial kits (Fortress Diagnostics Ltd, UK).

### 2.5. Analysis for Oxidative Stress Markers in the Kidney, Heart, and Liver Homogenates

**Superoxide dismutase (*SOD*) **activity was determined using the method described by Misra and Fridovich [14]. An aliquot of 0.2 mL of the homogenate was added to 2.5 mL of 0.05 M carbonate buffer (pH 10.2). The reaction was started by adding 0.3 mL of freshly prepared 0.3 mM adrenaline to the buffered sample mixture. This was quickly mixed by inversion and placed in the spectrophotometer. The reference cuvette contained 2.5 mL of the buffer, 0.3 mL of the substrate, and 0.2 mL distilled water. An increase in absorbance at 480 nm was monitored every 30 seconds for 150 seconds against blank.

SOD was calculated in units as the amount necessary to cause 50% inhibition of the oxidation of adrenaline to adrenochrome during one minute.

(1) SODunit/mg  of  protein=SODunit/mLproteinmg/mL×  dilution  factor

**Catalase (*CAT*)** activity was determined using the method described by Sinha [15]. In six different tubes containing increasing concentration of hydrogen peroxide (0 - 640 *μ*M); 2mL of acidified potassium dichromate (5%) was added. Each tube was shaken and heated at 100°C for 10 min, then allowed to cool and the optical density was read at 570 nm. A standard curve of absorbance against concentrations of hydrogen peroxide (H_2_O_2_) was then plotted. For the assay, 1 mL of homogenate was transferred to a tube containing 2mL of hydrogen peroxide (0.2 M) and 2.5 mL of 0.01 M phosphate buffer (pH 7.0) and mixed. From this mixture, 1 mL of solution was withdrawn after every 30 seconds and transferred into 2 mL of acidified potassium dichromate for 120 seconds. The mixture was shaken and heated for 10 min at 100°C. After cooling, the optical density was read at 570 nm against a blank.

The activity was then calculated in units of catalase as the amount required catalyzing the reduction of 1 *μ*M of H_2_O_2_ in 1 min.

**Lipid Peroxidation **was measured using the malondialdehyde (MDA) method as previously described by Biswas* et al*. [16]. In this assay, 0.4 mL of homogenate was added to a tube containing 2mL of acetic acid and 2mL of a working reagent (NaOH (0.5 N) + thiobarbituric acid). The mixtures were incubated in an oven at 100°C for 20 minutes; the absorbance was then read at 532 nm against the blank. The concentration of lipid peroxides was then calculated from the molar extinction coefficient of 1.56 × 10^5^ mole^−1^ cm^−1^ as follows:

(2) Concentration  of  MDA=ODLεε=1.56×105 mole−1 cm−1,

where *ε* is the molar extinction coefficient and* L *is the length of cuvette (cm).

**Concentration of reduced glutathione (*GSH*)** was carried out as described by Ellman [17]. In this assay 100 *μ*L of homogenate was added to the tube containing 1500 *μ*L of Ellman's working reagent (5 mg of 2,2-dithio-5,5-dibenzoic acid (DTNB) in 250 mL of phosphate buffer, pH 6.5). The mixture was shaken and incubated during 60 minutes at room temperature. The absorbance was then read at 412 nm against blank. The concentration of GSH was calculated from the molar coefficient of 13600 mole^−1^cm^−1^ as follows:

(3) $$\begin{array}{c} Concentration\;\;of\;\;GSH\left(\;mol/mg\;\;protein\;\right)=\varepsilon .L.C \end{array}$$

where *ε* is the molar extinction coefficient,* L* is the length of cuvette (in cm), and C is the concentration of GSH (in mM).

**Total Proteins** were estimated by the method previously described by Lowry* et al.* [18]. To different tubes containing separately 0.2 mL of homogenate and 0.2 mL bovine albumin (2 mg/dL) standard, 2 mL of distilled water and 1 mL of working reagent [1 vol (CuSO_4_ + NaK) + 100 vol (Na_2_CO_3_ + NaOH)] were added. The contents were mixed by inversion and incubated at room temperature for ten minutes. Absorbance was then read at 570 nm against a blank. The concentration of total proteins in the homogenate was determined from the equation below as

(4) Proteinmg/dL=concentration  of  standardOD  of  standard×OD  of  test  sample.

### 2.6. Histopathology of the Heart, Kidneys, and Liver of STZ Induced Diabetic Rats

On the last day (after 8 weeks), the animals were sacrificed and quickly dissected and small slices of liver, kidneys, and heart samples were fixed in 10% formalin and used for histopathological studies.

### 2.7. Statistical Analysis

Data was analyzed using SPSS 11.1 software. Variances were compared across groups using the one-way ANOVA and results were expressed as mean ± SD. The Waller-Duncan test was used to test for significant differences between means.* P* values of less than 0.05 were considered to be statistically significant.

## 3. Results

### 3.1. In Vivo Antioxidant Activity: Effect of C. afer on Streptozotocin-Diabetic Rats

**Figure 1:**
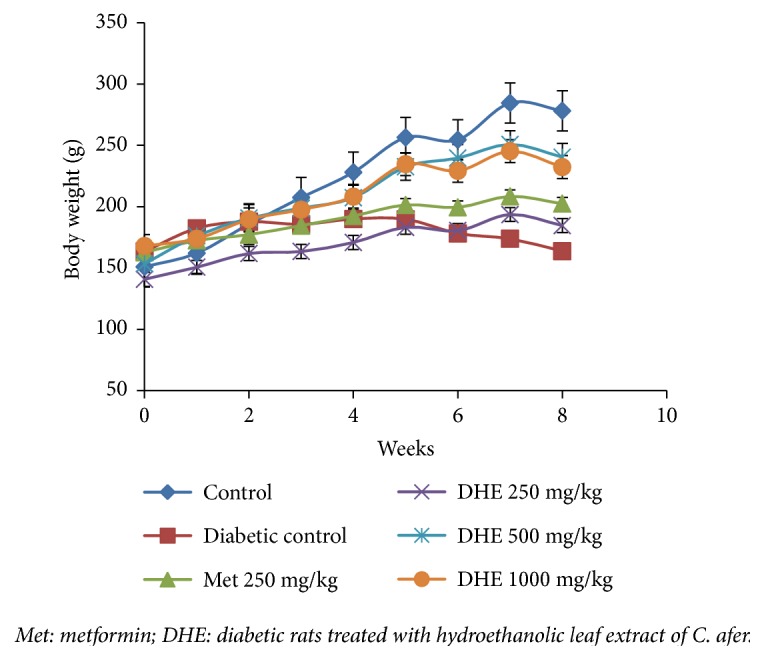
Body weight in streptozotocin-diabetic rats compared to normal rats.

*Effect on Physiological Parameters*. A significant decrease in body weight (p <0.05) of nontreated diabetic rats was observed in the groups treated with* C. afer* and metformin Figure 1. This decrease in weight of diabetic rats is an indication of physiopathology diabetes.

*Effect on Oxidative Stress Markers*. The effect of the hydroethanolic extract of* C. afer *on the hepatic, cardiac, and renal oxidative stress markers was analyzed and presented in Tables  1(a),  1(b), and  1(c).

Streptozotocin intoxication induced significant increases in the antioxidant enzymes activities (SOD, CAT) and lipid peroxidation product (MDA) (p< 0.05), as observed in the diabetic control rats. Administration of the* C. afer *extract (1000 mg/kg) significantly prevented (p< 0.05) streptozotocin effect on the SOD in the hepatic (35%), renal (78.5%), and cardiac (52%) tissues when compared to control diabetic rats. Metformin had the best inhibition effect on changes in antioxidant activity (hepatic 38.15%, renal 79.04%, and cardiac 56.2%) than* C. afer* extract. Similar results were observed with the catalase activity where the metformin administration (hepatic 77.18%, renal 72.88%, and cardiac 61.18%) and the extract (hepatic 72.07%, renal 72.70%, and cardiac 51.61%) prevent the increase in antioxidant enzyme activity. Generally it was observed that the antioxidant activities were higher in the cardiac tissue than in the hepatic and renal tissues.

The increase in antioxidant defense activity was accompanied by an increase in the lipid peroxidation, measured by malondialdehyde (MDA). Metformin and the plant extract at the dose of 1000 mg/kg significantly reduce (p < 0.05) the concentration of MDA, compared with control diabetic rats. The percentage reduction of the lipid peroxidation in the liver, heart, and kidney was about 82.3%, 67%, and 55.1% for the metformin and 85.7%, 73.2%, and 55% for* C. afer *(1000 mg/kg), respectively. The activity of the extract was higher than the reference, metformin.

The concentration of reduced glutathione (GSH) was significantly higher in the diabetic control group compared to normal control. Administration of extract and metformin significantly (p<0.05) prevented the increase of the glutathione concentration. Thus, the extract and metformin maintain the antioxidant status and prevent lipid peroxidation.

### 3.2. Effect of Hydroethanolic (HE) Leaf Extract of C. afer on Biochemical Parameters of Streptozotocin-Intoxicated Rats

The effects of streptozotocin on selected biochemical parameters revealed significant increases (p < 0.05) in ALT, AST, and urea level. Similarly, significant increases (p < 0.05) in ALP, LDH, creatinine (CRE), and bilirubin levels were observed in diabetic control group compared to the normal control group (Table 2). In diabetic rats treated with hydroethanolic extract of* C. afer* and metformin, we observed the improvements of these parameters towards the normal values in a dose-dependent manner when compared to the untreated group.

### 3.3. Effect of C. afer on Some Visceral Organs of Streptozotocin Intoxication in Rats (Histopathological Examination)

**Figure 2:**
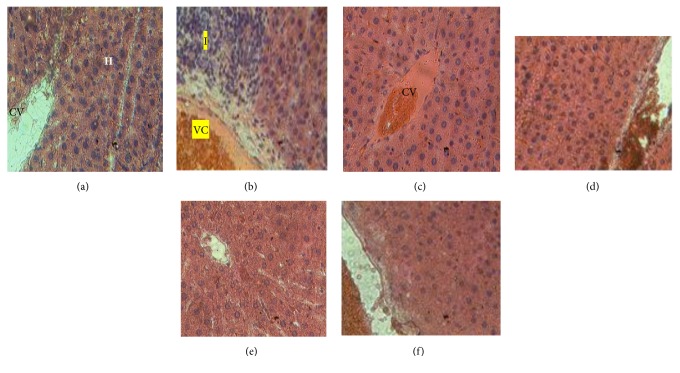
Histological examination of sections from the liver of experimental animals after 60 days of administration of the hydroethanolic leaf extract of* C. afer* showing abnormal architecture (b) compared to normal group (a).* CV: centrilobular vein. VC: vascular congestion. H: hepatocytes. I: leucocyte infiltration. (a) Control. (b) Diabetic control. (c) HE 250 mg/kg. (d) HE 500 mg/kg. (e) HE 1000 mg/kg. (f) Metformin.*

**Figure 3:**
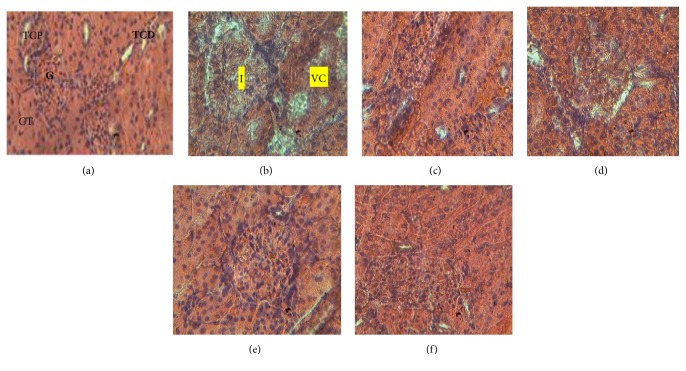
Histological examination of sections from the kidneys of experimental animals after 60 days of administration of the hydroethanolic leaf extract of* C. afer* showing abnormal architecture (b) compared to normal group (a).* TCP: proximal tube; TCD: distal tube; G: glomerular; CT: tubular clarification; I: glomerulosclerosis; VC: vascular congestion. (a) Normal control; (b) diabetic control; (c) diabetic treated with 250 mg/kg of C. afer; (d) diabetic treated with 500 mg/kg of C. afer; (e) diabetic treated with 1000 mg/kg of C. afer; (f) diabetic treated with 250 mg/kg of metformin.*

After 60 days of extract treatment, liver vascular congestion and leucocyte infiltration as well as kidney vascular congestion, glomerulosclerosis, and tubular clarification were observed in diabetic control (Figure 2) and rats treated with 250 mg/Kg (Figure 3). These findings demonstrate the involvement of diabetes in liver and kidney damage and the protective effect that the* C. afer* extract may have in this condition.

## 4. Discussion

Toxic agents' exposures have been reported to induce endogenous oxidative stress and tissue damage [19]. Streptozotocin (STZ) is a chemical agent that damages the DNA of the pancreatic islets through a free radical mechanism [20]. Hence it is commonly used for the induction of experimental diabetes in animals. Destruction of the pancreatic islets may impair glucose utilization and increased free radical generation due to insulin deficiency [21, 22]. Hence the implication of oxidative stress in diabetes is seen as either a causative factor or its consequence [21] and controlling oxidative stress may either help prevent the development of diabetes or control its consequence. The increase in free radical generation in STZ induced diabetes has been reported to decrease the activities of hepatic SOD and CAT in experimental rats [23, 24] and hamsters [25].

On the contrary, the present investigation revealed an increase in the antioxidant enzymes (CAT and SOD) activities in the kidney, liver, and heart tissue homogenates of STZ induced diabetes. This observation was not new since other groups of researchers have earlier reported similar results. Ramanathan* et al.* [26] in studying the antioxidant enzymes activities in the brain of streptozotocin diabetic animals observed increases in SOD and CAT activities, whereas glutathione peroxidase activity remained unchanged. Furthermore, Qujeq and Rezvani [27] reported an increase in erythrocyte catalase activity in STZ intoxicated rats. Streptozotocin has been equally reported to induce a distinct elevation in the activities of intestinal catalase (123.9%) and superoxide dismutase (71.9%) and a decline in the activity of glutathione peroxidase (67.7%) [28]. Similarly, STZ administration is known to induce significant increases on hepatic superoxide dismutase and catalase activities and a decrease in GSH-Px activity in diabetic rats [29]. Another group of researchers observed an increase in the activity of CAT in liver, heart, and blood with a decreased in kidney, while SOD activity was increased in liver, heart, and pancreas [30].

SOD and CAT function in a sequential cascade manner in the antioxidant defense system. SOD has been touted as one of the most important enzymes in the enzymatic antioxidant defense system. As an antioxidant enzyme, SOD catalyzes the removal of superoxide radical generated from the oxidation of a singlet oxygen species. The end product of SOD action is hydrogen peroxide which is an inhibitor of SOD if allowed to accumulate. Hydrogen peroxide is also a substrate for the production of hydroxyl radical through the Fenton reaction cycle. Hence this is the importance of CAT in the breakdown of hydrogen peroxide as it is formed to water and oxygen [31]. In this way, SOD and CAT function in protecting the cell from oxidative stress [32]. Hydroethanolic extract of* C. afer* boosts the antioxidant system by contributing phenolic compounds that scavenge the reactive oxygen species and hence reducing the oxidative stress, improving the defense mechanism, and preventing the increase in antioxidant enzyme activities. A similar effect was obtained when metformin was administered. Early studies have reported that* C afer* contain phenolic compounds with radical scavenging activity [12, 33] capable of preventing oxidative stress.

Oxidative stress has been associated with a decrease in tissue glucose uptake and insulin secretion in diabetic condition [34, 35]; meanwhile, lipid (MDA) and protein oxidation (PO) products accumulate [36]. This is in conformity with the results obtained in the present study characterized by a significant increase of MDA concentration in streptozotocin-diabetic untreated rats. This may be due to the fact that the hyperglycemic environment inhibited radical scavenging activity, exposed the lipids to oxidation Administration of* C. afer *extract, reduced the accumulation of glucose and contributed to the radical scavenging activity, and hence inhibited the oxidation of lipids. Glutathione (GSH) has a multifaceted role in antioxidant defense both as a direct scavenger of free radicals and as a cosubstrate for peroxide detoxification by glutathione peroxidases [37]; hence, a decrease in GSH concentration may be detrimental to the cell. STZ administration has been associated with a significant decrease in hepatic GSH concentrations [9] which may be due to decreased synthesis or increased degradation by oxidative stress [38]. In the present study, a significant elevation of hepatic and cardiac GSH level was observed in the untreated diabetic rats, but stable in the extract-treated diabetic rats when compared to control. This indicates that the extract reduces the oxidative stress leading to less degradation of GSH. All these observations could be explained by the potent antioxidant activity of* C. afer*.

Diabetic effect has been associated with weight loss and this effect was observed in STZ-induced diabetic rat [39]. During the 60-day experimental period, the body weight of STZ intoxicated rats reduced significantly, whereas there was a significant (p < 0.05) gain of body weight in rats receiving* C. afer* and metformin. Hence, administration of* C. afer* significantly (p < 0.05) to diabetic rats prevented the loss of body weight. The effect of* C afer* may be associated with stimulation of an increase in glucose metabolism, a mechanism of action suggested to plant extracts that reverse hyperglycemia [40].

Alteration of transaminases activities has been reported in diabetes. In the absence of insulin, amino acids serve as substrates in glycogenesis and ketogenesis and, hence, the activity of the transaminases [41]. An improvement in the levels of ALT, AST, and ALP was observed as a consequence of improved carbohydrate, fat, and protein metabolism due to administration of hydroethanolic extract of* C. afer*. The prevention of the alteration of ALT, AST, and ALP towards their normal levels may be due to the presence of flavonoids in the* C. afer* hydroethanolic extract, since they have earlier been reported to be hepatoprotective agents [42].

Diabetic complication was also observed on the deleterious effect on renal function characterized by the increases in plasma urea and creatinine concentrations. This effect has earlier been reported in streptozotocin induction of nephropathy in experimental rats [43, 44].* C. afer *modulates the deleterious effect of chronic diabetes and streptozotocin on the kidney of experimental rats by maintaining the creatinine and urea concentration towards normal values, suggesting a protective effect of the kidneys. Similar observations have been obtained when extracts of* Picrorhiza kurroa *and* Vernonia amygdalina *were administered to diabetic rats and this protective activity has been related to flavonoids [45, 46].

## 5. Conclusion

The results reported in the study confirm the protective effect of hydroethanolic leaf extract of* C. afer* against oxidative damage induced by STZ. This was achieved by enhancing the antioxidant defense system resulting in a decrease in the oxidative product. Regarding these results,* C. afer* appears to be an interesting medicinal plant and this highlights the investigation of* C. afer* as a promising source of antioxidant agents with protective properties.

## Figures and Tables

**Table:** (a) Hepato-antioxidant status of hydroethanolic extract of *C. afer* on STZ-induced diabetic rats

	**SOD (U/min/mg of protein)**	**CAT (U/min/mg of protein)**	**GSH (** ***μ*** **mol/mg of protein)**	**MDA (** ***μ*** **mol/mg of protein)**
**Control**	10.69 ± 1.63 ^a^	1.73 ± 1.22^a^	28.09 ± 9.93^a^	1.69 ± 0.35^a^
**Diabetic control**	22.91 ± 1.11 ^b^	9.99 ± 5.11^b^	44.12 ±14.73^b^	7.64 ± 0.26^b^
**DHE 250 mg/kg**	16.54 ± 1.85 ^a^	5.91 ± 4.36^a,b^	32.65 ±11.37^a,b^	2.92 ± 0.22^a^
**DHE 500 mg/Kg**	15.14 ± 1.42 ^a^	3.20 ± 3.02^a^	28.53 ± 7.23^a^	1.59 ± 0.25^a^
**DHE 1000 mg/kg**	14.91 ± 2.91 ^a^	2.79 ± 1.48^a^	24.56 ± 2.53^a^	1.09 ± 0.72^a^
**Met 250 mg/Kg**	14.17 ± 3.34 ^a^	2.28 ± 1.67^a^	23.24 ± 4.81^a^	1.35 ± 0.15^a^

**Table:** (b) Cardio-antioxidant status of hydroethanolic extract of *C. afer* on STZ-induced diabetic rats

	**SOD (U/min/mg of protein)**	**CAT (U/min/mg of protein)**	**GSH (** ***μ*** **mol/mg of protein**	**MDA (** ***μ*** **mol/mg de protein)**
**Control**	22.89 ± 4.10^a^	372.16±42.56^a^	237.94±5.52^a^	3.8 ± 0.36^a^
**Diabetic Control**	108.13±14.39^c^	983.24±65.17^c^	260.00±3.47^b^	6.49 ± 0.58^b^
**DHE 250 mg/kg**	40.45 ± 0.81^a,b^	531.91±41.55^b^	249.71±17.87^a^	3.05 ± 2.85^a^
**DHE 500 mg/Kg**	37.96 ± 7.41^a,b^	497.80±27.52^a,b^	240.15 ± 4.37^a^	2.60 ± 0.25^a^
**DHE 1000mg/kg**	23.26 ± 6.24^a^	475.82±68.50^a,b^	225.44±21.36^a^	1.74 ± 0.11^a^
**Met 250 mg/Kg**	22.66 ± 6.58^a^	381.73±37.67^a^	225.29±22.17^a^	2.14 ± 1.20^a^

**Table:** (c) Reno-antioxidant status of hydroethanolic extract of *C. afer* on STZ-induced diabetic rats

	**SOD** **(U/min/mg of protein)**	**CAT (U/min/mg of protein)**	**GSH (** ***μ*** **mol/mg of protein)**	**MDA (** ***μ*** **mol/mg of protein)**
**Control**	11.66 ± 2.13^a^	48.12 ±2.36^a^	133.97± 5.46^b^	4.68 ± 1.89^a,b^
**Diabetic Control**	28.85 ± 2.95^b^	186.67±78.46^c^	64.71± 10.59^a^	6.86 ± 0.33^b^
**DHE 250 mg/kg**	25.45 ± 2.93^a,b^	98.38 ± 4.77^b^	69.85 ± 3.33^a^	4.65 ± 0.46^a,b^
**DHE 500 mg/Kg**	20.16 ± 1.79^a,b^	83.38 ± 2.93^b^	87.65 ± 7.04^a,b^	3.94 ± 0.16^a^
**DHE 1000mg/kg**	13.95 ± 6.23^a^	50.96 ± 4.77^a^	125.00±2.89^b^	3.09 ± 0.59^a^
**Met 250 mg/Kg**	12.64 ± 1.28^a^	50.62 ± 9.04^a^	100.74 ±3.33^b^	3.08 ± 0.03^a^

DHE: diabetic treated with hydroethanolic extract of C. afer; Met: metformin. SOD: superoxide dismutase, CAT: catalase, GSH: reduced glutathione, MDA: Malondialdehyde. Each value represents mean ± S.E.M. (*n*=6). Means with different letters (a, b, c, and d) within a column are significantly different from each other at *p*< 0.05.

**Table 2:** Effect of *C. afer* on biochemical parameters in streptozotocin-diabetic rats.

**Biochemical** **Parameters**	**Normal** **Control**	**Diabetic** **Control**	**DHE 250 mg/Kg**	**DHE 500 mg/Kg**	**DHE 1000 mg/Kg**	**Metformin**
**ALT ** **(UI/L)**	76.92±6.68^a^	89.86±27.08^b*∗*^	76.46±5.13^a^	75.54±5.26^a^	74.92±4.32^a^	74.15±4.68^a^
**AST** **(UI/L)**	15.86±1.29^b^	19.89±2.21^c*∗*^	15.73±0.91^b^	15.43±0.52^b^	12.36±2.66^a^	12.48±1.73^a^
**ALP** **(UI/L)**	7.90±1.05^b^	15.15±1.23^a*∗*^	8.82±3.29^b^	7.35±1.13^b^	10.11±1.59^c^	10.84±2.19^c^
**LDH** **(UI/L)**	160±20.19^b^	212±31.14^c*∗*^	199±16.00^b^	191±69.41^b^	218±29.76^a^	175±48.73^b^
**CRE (mg/dL)**	1.01±0.14^c^	1.64±0.04^a^	1.23±0.15^b^	0.92±0.16^c^	0.83±0.04^d^	0.80 ±0.12^d^
**UREA (mg/dL)**	47.59±3.02^b^	70.97±17.92^c*∗*^	64.85±9.98^b.c^	43.75±13.87^b^	47.94±6.12^b^	25.85±2.98^a*∗*^
**BIL (mg/dL)**	2.33±0.61^a^	2.65±0.14^c^	1.98±0.26^c^	2.57±0.08^b^	2.68±0.13^b^	2.77±0.11^a^
**Total Protein (mg/dL)**	67.56±6.95^b^	70.55±4.54^c^	67.40±2.69^b^	63.75±3.17^b^	67.33±2.19^b^	53.41±13.67^a^

AST (Aspartate transaminase); ALT (Alanine transaminase); ALP (Alkaline phosphatase); LDH (lactate dehydrogenase); CRE (Creatinine); BIL (bilirubin total). The data represents the Mean ± SD for each group of rats. *n* = 6 (number of animals per group); *∗p*<0.05 = significant difference.

## Data Availability

All experimental procedures were carried out by the authors, and these have been described in the manuscript, with references being cited.
